# Human–machine coordination in mixed traffic as a problem of Meaningful Human Control

**DOI:** 10.1007/s00146-022-01605-w

**Published:** 2023-02-07

**Authors:** Giulio Mecacci, Simeon C. Calvert, Filippo Santoni de Sio

**Affiliations:** 1 Donders Institute for Brain, Cognition and Behaviour, Radboud University, Nijmegen, The Netherlands; 2 Department of Transport and Planning, Delft University of Technology, Delft, The Netherlands; 3 Department of Ethics and Philosophy of Technology, Delft University of Technology, Delft, The Netherlands

**Keywords:** Meaningful human control, Autonomous vehicles, Urban traffic, Mixed traffic

## Abstract

The urban traffic environment is characterized by the presence of a highly differentiated pool of users, including vulnerable ones. This makes vehicle automation particularly difficult to implement, as a safe coordination among those users is hard to achieve in such an open scenario. Different strategies have been proposed to address these coordination issues, but all of them have been found to be costly for they negatively affect a range of human values (e.g. safety, democracy, accountability…). In this paper, we claim that the negative value impacts entailed by each of these strategies can be interpreted as lack of what we call Meaningful Human Control over different parts of a sociotechnical system. We argue that Meaningful Human Control theory provides the conceptual tools to reduce those unwanted consequences, and show how “designing for meaningful human control” constitutes a valid strategy to address coordination issues. Furthermore, we showcase a possible application of this framework in a highly dynamic urban scenario, aiming to safeguard important values such as safety, democracy, individual autonomy, and accountability. Our meaningful human control framework offers a perspective on coordination issues that allows to keep human actors in control while minimizing the active, operational role of the drivers. This approach makes ultimately possible to promote a safe and responsible transition to full automation.

## Introduction

Automated driving systems (ADS) are increasingly widespread and will act as one of the most influential and enabling technologies of the forthcoming decades. Certain traffic environments, however, support vehicle automation better than others. Highways, for instance, are environments where the number and nature of the different actors is relatively predictable, while the degrees of freedom for vehicles are also limited and, in turn, the complexity of interactions are too. On the other extreme of the spectrum, the urban environment is characterized by the permeating presence of a broader variety of objects and actors, including vulnerable road users (e.g. pedestrians and cyclists, “VRU” hereafter), which explodes the number and complexity of interactions between vehicles, drivers and other road users. The relevance and unavoidability of this human component makes urban traffic particularly prone to a wide variety of human–machine coordination issues which can lead to safety and accountability problems. Most of the current studies on mixed traffic have focussed on the interaction between automated (“AV” hereafter) and human-driven vehicles (“HDV” hereafter) (Nyholm and Smids 2020; van Loon and Martens 2015). VRUs, such as pedestrians and cyclists, represent further elements to account for in an urban environment. Their superior freedom of movement further reduces predictability of their behaviour, and their vulnerability makes potential conflicting interactions with AVs particularly dangerous. Furthermore, it has been suggested that ill-intentioned pedestrians might exploit automated algorithms to their advantage or to simply stall the traffic (Calvert et al. 2016; Campbell et al. 2010; Millard-Ball 2018).

How should the (future) interaction between (partially) AVs, HDVs and VRUs be deigned and regulated? The problem is not merely technical, but also a normative one. We will follow Sven Nyholm and Jilles Smids in presenting three possible strategies that have been proposed to improve safety in mixed traffic and discuss the substantial value trade-offs (Nyholm and Smids 2020) that these strategies bring about. In fact, while promoting safety, other values may be compromised to an undesirable extent. For instance, according to Nyholm and Smids, programming AVs to behave more similarly to human drivers may make it easier for the latter to predict AVs behaviour. However, this may also result in programmers designing vehicles for the systematic infringements of some traffic regulations by AVs, which may raise other moral and legal concerns, for instance in terms of the democratic legitimacy of this shift of authority from the legislator to the designer. The general question that we investigate is how to reduce the value trade-offs raised by different design options, therefore minimising their negative impact and allowing to promote potentially conflicting moral goals (Van den Hoven et al. 2012).

Our claim is that the negative value impacts entailed by each of these strategies can be interpreted as lack of what we call Meaningful Human Control (MHC hereafter) over different parts of a sociotechnical system. We also argue that MHC theory provides the conceptual tools to minimize those unwanted consequences. We will show how “designing for MHC” constitutes a fourth alternative that is orthogonal to the three strategies proposed by Nyholm and Smids. Instead of simply asking whether vehicles should adapt to human behaviour or the other way around, we will focus on who should control which part of the system and what this means for the design of the system. By focusing on reducing control gaps, and by utilizing conceptual tools from MHC theory, our proposed approach helps to create a *context- and value-sensitive mixture of the different coordination strategies*. This promotes optimal coordination and, therefore, improved system safety. Our approach, however, does more than simply allowing a clever combination of these strategies: it reformulates them in terms of control problems that can be addressed by MHC theory in order to minimize a wide range of negative value impacts. Our approach, while preserving the coordination offered by a combination of the three strategies, also helps to safeguard ethical values such as democracy, individual autonomy, and accountability.

In line with this program, in the next sections we will present some known coordination issues in mixed traffic (Sect. 2) and three strategies that has been proposed to mitigate these issues (Sect. 3). We will, therefore, discuss some of the negative impacts that each of these strategies has on important ethical and societal values (Sect. 4). We will then show how reformulating the coordination issues in terms of MHC issues helps overcoming the value trade-offs that are brought about by the three different strategies (Sect. 5). Finally, we will discuss a case scenario to briefly exemplify how MHC could be used in urban traffic design (Sect. 6).

## Some human–machine coordination issues in mixed urban traffic

The urban traffic environment, being characterized by a wide variety of users, offers frequent occasions where not only HDV drivers, but also VRUs, have to coordinate their actions with AVs. One possible example is when pedestrians intend to cross the road, which regularly happens away from zebra crossings. Pedestrians rely on a number of behavioural cues to judge whether an incoming vehicle will slow down thereby allowing them to cross safely. HDVs drivers equally rely on behavioural cues when, especially in urban settings, have to cross their path with that of another vehicle, especially if those lack clear indications or signposting, such as are present at insertions.

To better understand the coordination issues at stake, we start by considering that there are at least two orders of problems. A first order of problems is due to the limited capacity that humans and automated systems have to understand each other’s intentions. These problems, discussed in Nyholm and Smids (2020), and studied by Van Loon and Martens (2015), affect all road users as well as AVs. In an urban scenario, VRUs, due to their superior freedom of movement and action, can further exacerbate this issue by performing a variety of limitedly predictable behaviours and, therefore, increase the complexity of the environment AVs would need to be able to deal with Nuñez Velasco et al. (2018). To complicate things even further, different levels of automation,^1^ and vehicles from different manufacturers, are likely to exhibit different driving styles. HDVs and VRUs will need to be developed and use multiple sets of expectation-forming processes. Different expectations on other actors’ behaviour should be entertained for a wide range of different road actors, from fully automated vehicles, through partially automated ones, to manual vehicles, bikes and pedestrians (Nuñez Velasco et al. 2018; Velasco et al. 2017). This is a heavy cognitive load for road users to deal with (Nyholm and Smids 2020; van Loon and Martens 2015; Wolf 2016), one that can potentially lead to miscoordination and consequent safety hazards. It has been proven extensively that inattention and distraction are almost unavoidable if cognitive load is too low (Louw et al. 2015; Regan et al. 2008; Sayer et al. 2005; Young et al. 2013), as a monitoring role for a driver or occupant of an automated vehicles places them unrealistically in a position they cannot properly control, which results in a loss of MHC (Calvert et al. 2020). Additional heavy cognitive loads also occur when transitions to manual control in automation are made (Eriksson and Stanton 2017; Merat et al. 2014; Varotto et al. 2018; Zeeb et al. 2015). The underlying point here is that while in practice, control can be assigned nominally, coordination and control from a meaningful human position may be unrealistically assigned beyond the cognitive ability of a human (Botvinick and Rosen 2009; Heikoop et al. 2018; Seeber 2011).

Coordination issues do not only create obvious safety risks, but also generate situations where it is hard to deem a user accountable for those cases where accidents might happen. A pedestrian that “safely” assumes that an automated vehicle will stop and let them cross, even in the absence of a zebra crossing, might find themselves surprised if the car were not to stop. This is somewhat analogue to what has been suggested to happen when drivers have to deal with automated vehicles. The way AVs are designed might produce for the users “perverse incentives”, thereby inviting behaviour that negates the primary goal of those who designed the technology, in our case traffic safety (Loh and Misselhorn 2019). Santoni de Sio and Van den Hoven (2018) suggested that, due to insufficient vehicle capabilities, overoptimistic beliefs promoted by car companies, or the nature of their cognitive capacities, human drivers of semi-automated cars might have a hard time complying with the (normative) expectations that car manufacturers and even the regulations may place on them. For instance, in low level AVs in which a driver is required to monitor the driving system, drivers may not be able to maintain the required level of attention on the operation of the vehicle, or take over operational control in time if requested by the system (Louw et al. 2015). We agree with the authors that a situation where a road user is not given a fair opportunity to discharge their obligations, whether it’s the driver or the crossing pedestrian, implies a diminished responsibility and blameworthiness in the case of an accident.

A second order of problems is not related to limited cognitive capacities, but to bad intentions. Authors have drawn attention to the potential intentional exploitation of automated traffic systems, especially in urban settings (Calvert et al. 2016; Campbell et al. 2010; Millard-Ball 2018)*.* An ill-intentioned user, once having identified a vehicle as automated, might take advantage of AVs’ typically risk-averse behaviour or overly predictable driving style to behave recklessly. This applies to drivers of HDVs as well as to VRUs. For example, VRUs may quickly get used to the fact that AVs are good at stopping and use that information to their advantage. They may even start relying on emergency braking and cross the road outside zebra crossings. This is not only undesirable, as it encourages illegal behaviour, but it also creates potentially very dangerous situations that can even lead to accidents.

## Common strategies to mitigate coordination problems

Nyholm and Smids (2020) discuss different strategies to mitigate the coordination issues indicated above. Their analysis is limited to coordination between HDVs and AVs but it is equally applicable to (and equally affects) a wider range of road users, including in our case VRUs. They categorise these strategies into three general classes:

1. Make AVs conform to, and imitate, HDVs’ behaviour, making their driving style less efficient and less strict in its rule following (Gerdes and Thornton 2016).
2. Make HDVs and VRUs conform to the AVs behaviour, further enforcing rule-abiding behaviour.
3. Avoid full automation in the vicinity of humans and rely on human input to resolve (some) coordination issues.

The first proposal suggests that we might design AVs to behave less “efficiently”, imitating human driving style. Reducing AVs’ “robotic”, unnatural behaviour, might improve coordination by reducing the gap between the expectations of HDVs and VRUs and the actual behaviour of an AV. A driver of an HDV, for instance, may feel stuck behind an AV that abides by the speed limits and that may subjectively appear unreasonably low, and attempt a dangerous overtaking manoeuvre. Programming the AV in such a way that it can in some cases break traffic regulations^2^ may help following drivers to feel more at ease. Pedestrians and other VRUs that expect human-like behaviour would be more careful and double check the speed of the oncoming vehicle, to make sure it intends to stop. Humanizing AVs driving style may also contribute to a reduction in intentional miscoordination, namely the exploitation of the automated components of traffic. An aggressive driving style, more similar to that of a human, could in some cases contribute to discourage people from exploiting the system to their advantage and even help with understanding what the intentions of the vehicle are. Revving, or hinting at movements, may be suggestive of certain intentions and prevent ill-intentioned pedestrians from slowing down the traffic flow by, for instance, loitering within, or in the vicinity of, the crossing area. Different behavioural cues, inspired to human intentional behaviour, such as displaying insecurity, could warn the road users and invite them to be careful. This is not a new suggestion, and it has been studied previously in the field of robotics (Van den Brule et al. 2016).

The second suggestion to improve coordination is to make humans behave more similarly to automated systems. Implementing alcohol interlocks, speed limiters, or any other form of imposed assistance, would probably result in an overall safer traffic environment. This strategy, originally proposed in the mixed traffic debate to address intervehicle coordination issues, is harder to implement in a genuinely urban setting where there is a predominant presence of VRUs. Besides the relatively obvious option of simply regulating behaviour more thoroughly (i.e. via traffic regulations), other measures may be possible, such as using wearables to “buzz” VRUs to raise their awareness of risks or nudge them towards a certain more desirable behaviour. Current research (Kayukawa et al. 2019; Montuwy et al. 2019) shows that this is not too far from large scale feasibility. A VRU could utilize information about incoming traffic or monitor the safety level of their current location or behaviour. This would allow them to be constantly more aware of themselves and the surrounding traffic. Ill-intentioned individuals may be reminded of their illegal behaviour and encouraged to act lawfully.

Finally, the third suggestion would be to stop pursuing full automation and settle for an optimally designed cooperative human–machine interface, at least as far as the urban environment is concerned. This would imply that coordination-difficult situations would be programmatically addressed by relinquishing operational control of the vehicle to the human driver (to the extent they are able to do so). Under normal circumstances, drivers would take advantage of automated driving functions. The advantages of this strategy, which to some extent resembles the current state of play, would be to grant better accountability in case of accidents and, in some particularly complex scenarios, improve safety. It is worth noticing that this proposal is not necessarily as trivial as it might seem. Despite raising some concerns in e.g. big tech corporates, of hindering innovation (as the old adagio goes: more control, less automation), it resonates with the position of several popular authors (see e.g. Mindell 2015; Pasquale 2020). They suggest that we should invest our best efforts in realizing an optimal human–machine interaction, rather than focusing on eliminating the human role in its entirety. Reintroducing human judgement in the coordination activity would in our case also additionally partially address the problem of intentional disruptions of the traffic flow.

## Ethical and societal implications of the three strategies

Each of the three strategies discussed in the previous section has its own merits and problems in terms of efficacy and feasibility. In this paper, we do not want to delve further into these aspects, which are better suited to a technical and/or legal approach. Rather, we will focus on the ethical and societal implications that might ensue from each of the strategies. In this section, we will still base our analysis on Nyholm and Smids’, and complement it with additional insights where needed.

Strategy 1: adapting automated behaviour to human behaviour, a.k.a. designing for imitation

Regarding the first of the proposed strategies, i.e. making automated systems behave more similarly to humans and in so doing “designing for imitation”, some ethical implications have been pointed out. Adopting such a strategy implies allowing the AVs to *systematically* break or bend laws, at least to the extent that this is what human drivers as a matter of fact do. If for instance AVs learn that human drivers usually do not respect the speed limit on certain stretches of road, so won’t they. This is ethically problematic as a matter of principle, insofar as traffic regulations were indirectly democratically approved. In turn, designers should not replace legislators and policy-makers in a de facto unilaterally decided legalisation of (some) programmatic rule-breaking, no matter how common these are. Also, traffic regulations were designed with safety considerations in mind, and increasing non-compliance by design may create unwanted safety risks (Smids 2018). Additionally, allowing by design AVs to behave like humans is a bad idea as it may be insufficiently sensitive to context and not sufficiently responsive to underlying moral and legal reasoning.

Indeed, *some kinds of* rule-breaking behaviour might be acceptable and even desirable. A good example of this, is the performance of prima facie illegal manoeuvres to enhance safety, such as swerving over a solid line to avoid a collision, or stopping at the side of a road to let an emergency vehicle pass. However, AVs of the foreseeable future may simply not have the moral sensitivity required to judge when an infraction of a traffic regulation is, all things considered, justifiable and even recommendable.^3^ Relatedly, this would also raise concerns about moral accountability and responsibility for those systems’ behaviour. Whenever a human driver breaks or bends a rule in the interest of avoiding what they consider to be a greater harm, they may be asked to justify their behaviour in front of a judge. This has the purpose of assessing whether this was, all things considered, a reasonable choice and, therefore, legally acceptable behaviour under the doctrine of necessity (Santoni de Sio 2017). It is debatable whether similar mechanisms of accountability and responsibility would be feasible in relation to intelligent machines.^4^ Finally, even assuming that design for imitation may make machines’ behaviour sometimes, even often, more predictable (hence allowing for better coordination), a common concern is that machine learning might also sometimes result in unpredictable behaviour, which would add yet another source of undesired safety risks.

Therefore, to summarize, a system that is optimized for coordination and human resemblance, if on one hand could make coordination safer, might raise some other serious safety issues, in addition to issues of democratic justification, accountability and responsibility.

Strategy 2: adapting human behaviour to automated behaviour, a.k.a. designing for lawfulness

The second strategy is to adapt the behaviour of human users to a machine behaviour that is highly standardized and therefore highly predictable. Though being per se a valid option to improve safety, Nyholm and Smids raised concerns about freedom and human dignity in the context of externally regulating human decision making in traffic. Technologies like speed limiting devices and alcohol interlocks may be effective measures to regulate drivers’ behaviour, but they might be perceived as a limitation to the freedom of choosing your driving speed or even to whether or not to obey the law in general (Smids 2018).

If freedom and dignity are a concern with regard to drivers, other road users may be even more gravely affected by these solutions. This may also raise legal issues. The Horizon 2020 Commission Expert Group in their recent report on the “Ethics of Connected and Automated Vehicles”, states that, “in line with the principle of justice, in order to address current and historic inequalities of road safety, [AVs] may be required to behave differently around some categories of road users, e.g. pedestrians or cyclists, so as to grant them the same level of protection as other road users. [AVs] should, among other things, adapt their behaviour around vulnerable road users instead of expecting these users to adapt to the (new) dangers of the road”. Requesting VRUs to adapt their behaviour to that of AVs could negatively affect a just distribution of burdens across the different road users.

Furthermore, technical solutions like wearable technologies supporting VRUs risk to be slippery slopes towards dystopic totalitarian scenarios where not only privacy but freedom itself may also be heavily compromised (Reiman 1995). Similarly to vehicle based speed limiting technologies, wearable electronics aimed to regulate human behaviour can negatively affect people’s autonomy and freedom of action. If compared to current forms of urban traffic regulation, such as traffic laws and law-supporting devices like traffic lights and zebra crossings, adopting specific assistive devices may nudge behaviour in a more direct way. This may further exacerbate the discomfort and ethical concerns that were raised with regard to drivers. In addition, the imposition of such restrictions on VRUs may be also considered as unfair. Unlike AVs users, VRUs do not have any choice about the introduction of automated vehicle on the road, and they may be put in a position to decide between sacrificing their own safety or their freedom, in the interest of the freedom of AVs users. Finally, though privacy may not represent a qualitatively novel problem, especially given the increasing spread of data collecting wearable technologies, we can see how certain aspects of implementing these devices in traffic regulations may contribute to exacerbate the problem. On the one hand, the amount of geographical and behavioural data that these technologies should be allowed to process in order to be effective would be substantially superior and more accurate than the current situation. On the other hand, the compulsory nature of their use, which would probably need to be regulated by traffic law in order to achieve some acceptable level of efficacy, could limit the effective capacity of VRUs to refuse consent to the processing and eventual retention of certain personal data. Comparable dynamics and ethical implications have been recently discussed with regard to COVID-19 tracing technology (Braithwaite et al. 2020).

Strategy 3: settling for a well optimized partial automation, a.k.a. designing for operational control

The third strategy discussed by Nyholm and Smids is to keep humans involved in the automated decision making. While promoting better coordination amongst different kinds of vehicles and between vehicles and VRUs, this strategy could create coordination issues at a lower level of human–machine interface, e.g. between drivers and their (partially) automated vehicles. The problem of transition of control (Calvert et al. 2020; Parasuraman and Riley 1997), one of today’s biggest challenges in partial driving automation(Varotto et al. 2018; Tillema et al. 2017; Vlakveld 2016; Merat et al. 2014), has been for several experts a valid argument to choose full automation over certain varieties of human machine cooperation and partial automation (Stanton and Marsden 1996). Humans have limited physical and cognitive capacities that should be correctly accounted for while designing interfaces (Carsten and Martens 2019). This is indeed a technical problem, but with important ethical implications. On the technical side, there are promising ideas on the horizon to partially address these challenges. For instance, cognitive roboticists have proposed different forms of cooperative control aimed to minimize transitions and to allow a fluid interaction between controllers and controlled AVs (Flemisch et al. 2019; Pacaux-Lemoine and Flemisch 2019). On the ethical side, failure to obtain satisfactory human machine coordination through technical solutions could result in what has been called moral (Danaher 2016) and legal (Santoni de Sio and Mecacci 2021) scapegoating. AVs manufacturers could rely on legal contracts and hardly fulfillable clauses to transfer their responsibility to the users, for instance requiring constant system monitoring in situations where it is arguably difficult to perform such task. Finally, this strategy may ultimately defy the purpose of–fully–automating driving tasks, which is to relieve drivers from their active involvement, and free the traveling time for rest, productivity or leisure (Table 1).

**Table 1:** Main negative value impacts of the three proposed strategies

	Strategy 1. Designing for imitation	Strategy 2. Designing for lawfulness	Strategy 3. Designing for operational control
Values negatively impacted	Safety Democracy Accountability/responsibility	Autonomy Dignity Freedom Justice Privacy	Innovation Safety Productivity/leisure

## Ethics of “mixed traffic” and meaningful human control

It seems so far that we are stuck with a set of options, none of which looks very satisfactory from an ethical point of view. In this second part of the paper, we claim that these three strategies do not help (alone) carving the problem at the right joints. While mainly striving to preserve safety through coordination (and as we have seen, with some caveats), they negatively impact a number of other values that are also societally important.

We will claim that a different approach could promote safety while better safeguarding other values. Namely, instead of formulating the mixed traffic problem in terms of a coordination problem, we propose to frame it in terms of a (moral) control problem (Sect. 5.1). This is not to detract from the research in coordination, which remains perfectly relevant, but it is to show how an MHC perspective could help achieve safety (and to a reasonable extent, coordination) *through control*. It is not, in other words, a problem of *who/what coordinates with whom/what*, or *whether there should be full automation* in the first place, but rather a matter of *who should be in control of a system, when and in which sense*. We will show that the manifold negative value impacts can be fruitfully interpreted as (partially) due to insufficient—or suboptimally distributed—human moral control across the system at large. Once we can see that, a normative theory of control such as MHC becomes relevant. In that regard, we will show how MHC provides a useful framework to operationalize, quantify and understand moral control, and work towards its optimal distribution (Sect. 5.2). This will open up the way for a fourth strategy in achieving coordination and, ultimately, safety, “designing for MHC” (Sect. 6).

### Why the three strategies’ problems are actually (moral) control problems

We claimed that designing for imitation (strategy 1) may increase human machine coordination and address some safety issues but it might at the same time create other safety risks while creating potential safety hazards and negative implications for democracy and accountability. Designing for lawfulness (strategy 2), while being indeed in line with the values of democracy and responsibility, may not address some safety risks in human machine coordination; at the same time, it might also have a potential negative impact on human freedom, dignity and justice. Keeping human drivers in the loop for harder decisions (strategy 3), while partially addressing the problems generated by the other two, would compromise on a main purpose of driving automation, which is to relieve human drivers from their driving tasks. Also, and perhaps more importantly, it would introduce potential safety hazards due to well-known human factor problems concerning transitions of control.

This is a classic case of what, in the value-sensitive design theory, has been called a problem of moral overload (Van den Hoven et al. 2012), a situation where we are “confronted with a choice situation in which different obligations apply, but in which it is not possible to fulfil all these obligations simultaneously”. Value-sensitive technical innovation has been suggested to gradually reduce the trade-offs that a certain moral overload situation requires us to make (van den Hoven 2013). In a similar fashion, we suggest that the value trade-offs brought about by the three strategies might be partially overcome by identifying the lack of (moral) control as one of their causes. Improving that kind of control, we will claim, would relax some of the value trade-offs.

*Designing for imitation* is a strategy that proposes to allow technology to break and bend traffic regulations *in an uncontrolled way*. As noted above, human drivers are also sometimes allowed to break traffic regulations, but only to the extent that: a) they do so out of situational necessity, that is to avoid a substantial risk in a context in which no other legal means are reasonably available; b) they do so within the limits of proportionality, that is the regulation violation is not more serious than requested to avoid the risk; c) they remain accountable for their behaviour, that is they are able and willing, if so required, to explain their reasons to public institutions that can assess the reasonableness of the behaviour and sanction it if needed. The availability of sufficient reasons to break a regulation that was produced in a democratic process is what may make the rule-breaking eventually justified. Borrowing the language of one influential theory of moral responsibility, we may say that it is this “responsiveness to the relevant reasons” that makes human drivers better *moral* controllers and therefore fairer recipients of moral and legal accountability and responsibility than machines (Fischer and Ravizza 1998). We are not thereby implying that AVs will never be able to achieve such a flexible responsiveness to (moral) context. Advances in AI might in fact bring significant developments in that respect. What we are claiming is that the extent to which the design for imitation strategy brings about ethical concerns crucially depends on this responsiveness. At a more general level, we have noticed that this strategy is also problematic from the point of view of democracy. In fact, it would allow technology producers to legalise existing widespread violations of traffic norms by designing vehicles that imitate human behaviour. This is a problem for democracy insofar as technology producers are not subject to public control and so they do not have the moral and legal authority to make these choices.^5^ Borrowing the language of one influential theorist of liberal democracy, these decisions are illegitimate insofar as they may not sufficiently “track the interests and ideas of ordinary people” (Pettit 1999, p. 11).

The second strategy, i.e. *designing for lawfulness*, proposes to have VRU and HDV adapt to AVs’ robotic behaviour, and its value impacts are also interpretable under a (lack of) control perspective. In fact, what is compromised in that case is the road users’ capacity to exercise decision-making power, and therefore, to *remain in moral control of their own behaviour* by having it depending on their understanding and interpretation of the regulations, as opposed to mechanically complying with them. It might even be that here human control is not diminished, but its locus rather shifted towards a supraindividual agent, such as e.g. the state as regulator or the designers of the technology. However, that is precisely why individual moral autonomy, freedom, dignity and justice are potentially affected: (some) persons are (partially) deprived of their moral control over their actions. Both strategy one and two are thus characterized by a reduction of moral control, or an (undesirable) shift thereof.

**Fig. 1:**
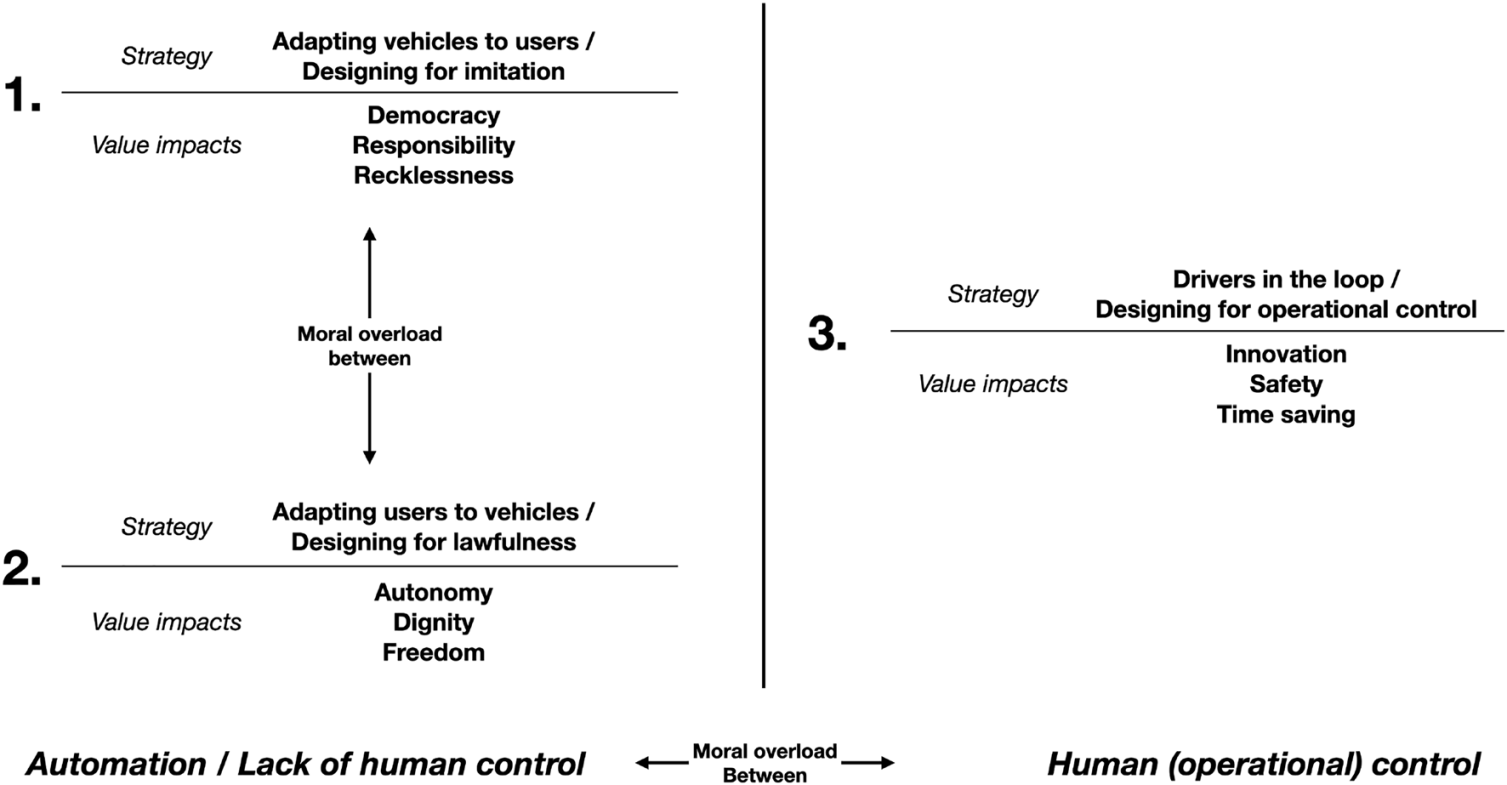
The reciprocal relations among the three strategies

We further suggest that these two strategies can be grouped under one horn of a more fundamental control dilemma, the one between full and partial automation. The full automation horn of this dilemma is the one on which strategy 1 and 2 are grounded (see Fig. 1). They both suffer from ethical issues that are ultimately due to a reduction or shift of moral control, either by drivers (or users) on their vehicles or by some VRUs on their own behaviour. The latter horn consists in strategy 3, which brings back (some) human control to drivers (and, indirectly, VRUs), but it does so in the form of *direct, operational control*, thereby resulting in safety concerns and a suboptimal state of technological slowdown. More importantly for our argument, this operational control may not amount to that moral control which partly constitutes human autonomy and ground moral responsibility, which in the rest of the paper we will call: Meaningful Human Control.

### Meaningful human control—background

The notion of Meaningful Human Control finds its roots in the political debate about the so-called “killer robots” in warfare (lethal autonomous weapon systems) (Horowitz and Scharre 2015; Moyes 2016). The general idea was to require a more genuine form of human control over autonomous machines that operate in conditions where stakes are very high. It is common in automated warfare to simply require an operator to be “in the kill chain” to preserve control and responsibility over highly automated technical systems (Wenzl 2018). However, this requirement has been deemed to be excessively loose since the operator is typically subject to a number of difficulties inherent to the interaction with intelligent systems. These are commonly cognitive limitations regarding the amount of knowledge one is provided by the system, the short time in which decision making has to occur, and biases that can be induced by a certain way of presenting information (Schwarz 2018). Human agents are often requested to make decisions without having a complete picture of both the situation and the potential consequences. This would take a long time, which is precisely why artificial intelligence systems are deployed to assist the human controller in the first place. Furthermore, humans are naturally lazy, as they tend to lean towards passively accepting decisions and actions that are provided to them by automated systems or even the very surrounding environment (Haselager et al. 2008). To summarize, simply sitting in the “war room” may not grant sufficient control over the outcomes of automation assisted operations. The limited amount of control, together with the dynamic intelligence and sometimes inscrutability of the automated part of the decision making, creates in turn accountability problems.

On one hand, designated controllers have justifiably diminished awareness of, and causal contribution to, the decision-making process. On the other hand, external agents that are called to express a moral or legal judgement over the actions of a system, encounter difficulty in determining how moral and legal responsibility should be distributed across the different elements of the system, one of those being the human agent(s). These (partial) voids of accountability and moral responsibility in automated sociotechnical systems have been called “responsibility gaps” (Matthias 2004; Santoni de Sio and Mecacci 2021).

The notion of MHC has been recently further substantiated by Santoni de Sio and Van den Hoven (2018), who provided a philosophical account. Inspired by the debate on free will and moral responsibility, and in particular by Fischer and Ravizza’s (1998) account of “guidance control”, they proposed two main conditions for MHC over (partially) autonomous systems.^6^ Those conditions are meant to provide an ideal conceptual ground to assess and design for control where human agents are interfacing with intelligent systems. There are two main requirements for MHC, called “tracking” and “tracing”.

Tracking regards the extent–and quality–to which a system’s behaviour is able to “track” a human controller’s (moral) reasons to act. These can be anything that motivates and/or explains an action. An action, as opposed to a simple movement, is performed according to a reason, e.g. intentions, goals, desires or even something more intersubjectively shared, like values and norms.^7^ In this particular context, we are mostly interested in those reasons that have a moral connotation, because they allow us to evaluate an agent’s conduct and keep them responsible. Tracking these moral reasons implies in simpler terms the ability of an automated system to achieve a stable alignment between its behaviour and the–constantly changing–human intentions and values.

Tracing concerns the extent to which, for any given control scenario, one or more human controllers can be identified at some point of the technological design or deployment context. The requirement further prescribes that, for a controller to count as such, they have to have a sufficient degree of knowledge of the system and, if required by the specific system’s design, the capacity to steer its behaviour. Also, such controllers have to be morally aware of their role in controlling the system, thereby recognizing themselves as morally responsible for the (negative) consequences of the system’s actions. In other words, they have to be aware that they will be deemed morally responsible for the actions of the system they are meant to control.

As further clarified by Mecacci and Santoni de Sio (2020), MHC, as opposed to classic notions of control in engineering and (traffic) psychology, and in virtue of its different (normative) requirements, applies to a wider range of controllers for any given system. MHC is not grounded on a direct causal intervention, i.e. on an agent directly acting upon something, but rather on a rational one, i.e. on an agent (or a group of agents) expressing their reasons to influence and guide another (artificial) agent. Moreover, expressing human agents’ reasons can be done in many different ways, the most common of which could be producing a certain intelligent system while taking certain societal goals and values clearly in consideration during all the design stages. This makes it possible to deem human agents in control of artificially intelligent systems by merely having those systems reliably aligned to their (moral) reasons. This means, in turn, that the different participation of a wide variety of agents in controlling the system can be appreciated. These agents need not be in a direct relationship with–nor do their actions need to be in temporal contiguity to–the *re*actions of a system. For instance, if the tracking condition is realised, an engineer that developed a certain piece of software, or a policymaker that regulated its deployment, might be recognized, within the MHC framework, to exercise control on a system’s behaviour, and therefore to be (partially) responsible for its actions.

The rationale behind the notion of MHC is twofold: autonomous machines should remain under some human moral control, even while they are acting on their own accord; and human moral responsibility for the machine behaviour should be maintained. MHC is a kind of control that expresses the ideal conditions under which a human agent could, and should, remain in moral control and, therefore, retain moral responsibility for a certain machine’s behaviour. These conditions can be fulfilled to reasonably high degrees even in those high or full automation scenarios where operational control is not possible by design. Finally, by setting requirements on human controllers’ knowledge and capacities, MHC does not only support responsibility, but inherently promotes a safer interaction between controller(s) and controlled systems.

The notion of MHC has been further operationalized by spelling out the components that are part of the two conditions and proposing applications for the concept (Calvert et al. 2019; Calvert and Mecacci 2020; Mecacci and Santoni de Sio 2020). This was in order to facilitate both the assessment of meaningful human control and the design of machines and infrastructures according to MHC requirements and guidelines. In particular, the notion of tracking has been enriched by the means of the so-called “proximity scale of reasons” (Mecacci and Santoni de Sio 2020). This scale, inspired by both philosophy of action (Bratman 1987; Raz 1975) and traffic psychology (Michon 1985), proposes the use of a particular unit of measurement, “proximity”. This unit helps measuring how directly different agents’ reasons to act (not just intentions, but overarching goals, interests and values) influence the behaviour of an intelligent system. It will become clearer in the remainder that the proximity value is roughly meant as an abstract, and fairly normative, unit of measurement to help identifying a range of human controllers and the degree of their involvement in, and responsibility for, a certain automated behaviour. The nature of this “influence” is a philosophically complex subject that should nonetheless be at least preliminarily discussed here.

**Fig. 2:**
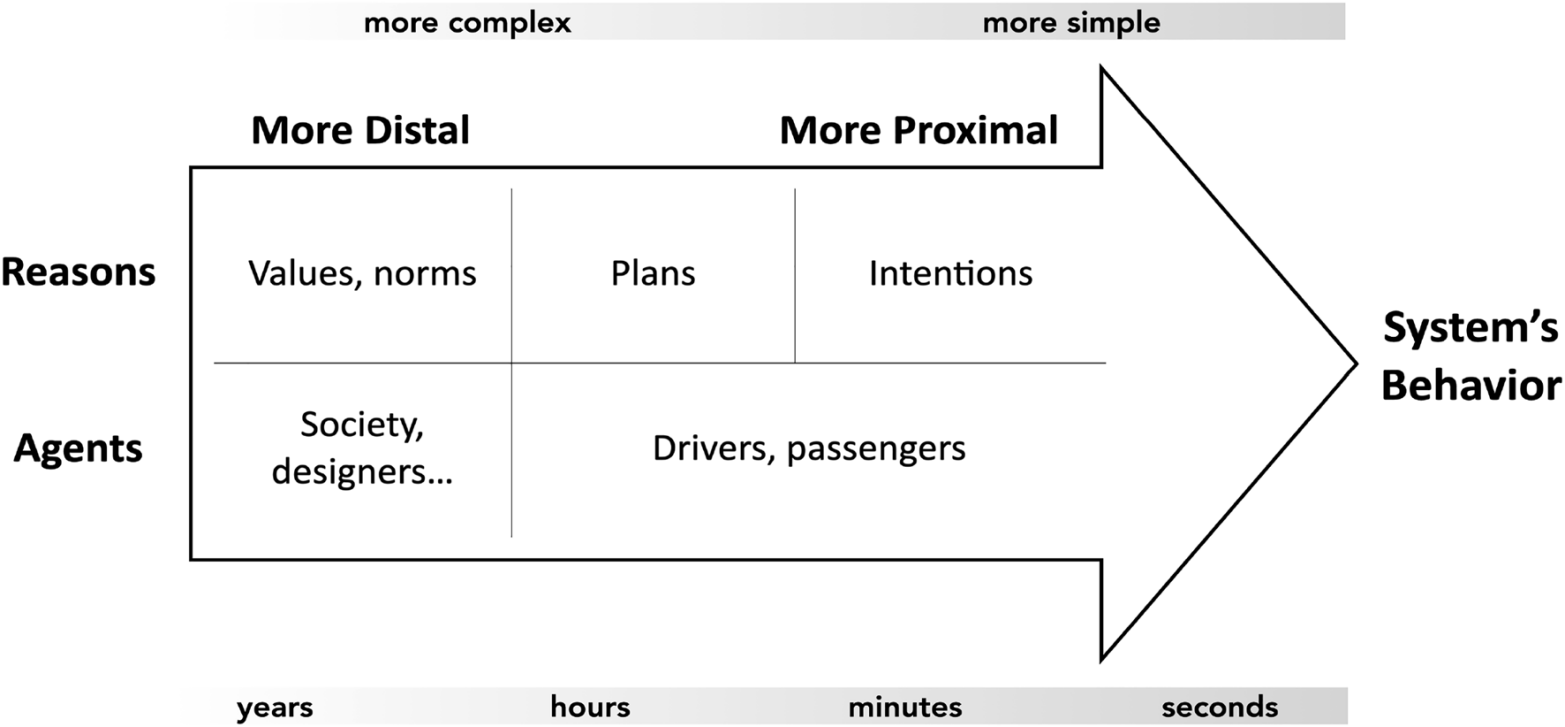
The proximity scale of reasons in the case of automated mobility. This scale is a continuous space where different reasons to act can be visualized in their reciprocal relation and in relation to their influence on a system’s behaviour (Mecacci and Santoni de Sio 2020). Different reasons can be attributed to different typical agents in a system. Those reported in the pictures have mainly exemplificatory purpose

The tracking criterion, as mentioned, aims at assessing a relation between a controller and a controlled (part of the) system. This relation is not exclusively an operational, causal relation, as it needs to accommodate for forms of control directed towards highly autonomous systems, where the causal influence between controller and the controlled system can be extremely weak. For instance, a designer of a technology might fulfil the MHC criteria for control rather well, while at the same time, being very loosely related, in causal terms, to a certain behaviour of a certain system they designed. Therefore, this “influence” is not meant to be (just) a causal one. Rather, as it is traditional in part of the history of philosophy of action, the “influence” is also evaluated in terms of how directly (in terms of complexity and time frame) a certain reason “explains” a certain behaviour and makes it understandable. Complex reasons, e.g. general plans or goals, can be decomposed into simpler atomic intentions, and they tend to influence and explain a system’s given behaviour over a longer timeframe (Fig. 2). For instance, an agent might be riding in an AV because they want to go home. This reason is relatively more general and abstract than, for instance, the reason to take a particular route, and not another. Both those reasons influence action but they do so at a different level of proximity, the latter being more proximal to the actual behaviour of the automated system. In other words, more distal reasons can be instantiated by a wider range of different courses of action, therefore, granting more autonomy to the very system, but without necessarily diminishing a human agent’s control (this is explained in detail in (Mecacci and Santoni de Sio 2020)).

## Designing for Meaningful Human Control

In this section, we will argue that “designing for MHC” is a viable fourth strategy that crosses the other three strategies diagonally. By striving to maximize MHC across all the elements of a traffic system, we can achieve high levels of safety and coordination while minimizing the value trade-offs and the negative impacts that each of the three discussed strategies brings about. As we have shown above, those negative impacts can be reformulated due to insufficient (moral) control. Simply switching from one strategy to another, even cleverly, can certainly grant optimal safety and coordination, but leaves the other value impacts insufficiently addressed. There is always one agent or other that pays a price. This is the consequence of those trade-offs we discussed. MHC theory, however, strives to find ways to keep the whole system in control, rather than simply trading it across its users. This is because control is based on the two conditions of “tracking” and “tracing”. Therefore, for as long as a system–as a whole–responds to one or more agents’ reasons (tracking), and those agents meet certain requirements (tracing), then the whole system can be deemed under control of those agents. This is even if those agents are not actively doing anything. Hence, to the extent these criteria for MHC are achieved, the negative value impacts that were due to lack of control can be addressed.

### How MHC can help overcoming the trade-off between automation and human (operational) control

One of the fundamental tenets of MHC theory is that the dichotomy between automated control and human control is a false one. As we have seen in Sect. 5, MHC theory frames the notion of control in more abstract terms that: (a) go beyond the direct, operational control of an operator or system (tracking) and (b) explicitly require that some agent(s) along the chain have sufficient technical and moral competence to bear moral responsibility for the behaviour of the system (tracing). The conditions for control considered by MHC theory support the idea that fully autonomous systems might be deemed to respond to a set of intentions and values that pertain to human agents who are distant in time and space from the moment a certain behaviour is displayed, and that these may nonetheless remain responsible for the system behaviour. This is despite the absence of a clear and direct causal connection between the controller and the controlled system. In that regard, MHC provides some indications to identify “distal” actors that, in virtue of their role in the chain of production, deployment and use of a certain technology, can be considered among the controllers of a system, as well as bearing (partial) responsibility for its behaviour. The theory partially relieves us from some of the costs of full automation—i.e. the fact that automated systems’ behaviour might be not clearly responding to any clearly recognisable human agent’s reasoning, unaccountable or generate “responsibility gaps”. In turn, this allows us to more fully reap the benefits of automation.

The fact that automation and control can be compatible does not mean that operational control, which constitutes the main proposal of strategy 3, should be entirely discarded. Automation technology commonly strives to reduce the role of the agent that is operationally in control of a system as much as possible, and MHC theory promotes human involvement in only those operations humans are genuinely suitable for. It promotes human factors investigation of those roles. All in all, bringing the driver back in the loop might in some cases be the best option, but that should be done with the purpose of designing for MHC, and should only be considered when the MHC conditions for control cannot be better fulfilled otherwise. This means more attention to safer transitions of control and greater possibilities to delegate more and more tasks to automation, thanks to the clearer ways to attribute responsibility to distal agents. Ultimately, this approach promotes and safeguards innovation.

### How MHC can overcome the trade-off between designing for imitation and designing for lawfulness

A similar story is also applicable for the two strategies that constitute the full automation scenarios where we observe that burdening value trade-offs are brought about by the loss of human control. And again, the negative value impacts of these two strategies, both promising avenues to minimize coordination problems and promote safety, can be minimized by maintaining a high degree of MHC across the whole system. Retaining MHC means keeping the behaviour of AVs responsive to the relevant human actors’ intentions (tracking) as well as keeping (some) human agents able and motivated to discharge their moral accountability and responsibility for the lawful or reckless behaviour of the vehicles (tracing). It also means that democratic control can, to a certain extent, be exercised and assessed, as democratic values are put among the possible sources of autonomous systems’ actions by the tracking scale of reasons (Mecacci and Santoni de Sio 2020). As we have mentioned, the problem with democracy is mainly present in those cases in which we design to systematically infringe traffic regulations, i.e. “designing for imitation”. In this case, the designers are in a way illegitimately replacing the legislator by deciding to exempt some vehicle from rule-abiding. If rather, a human agent could be meaningfully identified as a controller when the need arises for automated vehicles to infringe the law, these cases may be treated more similarly to those already numerous cases of human recklessness, if and when that is deemed to be necessary. Autonomy, freedom and human dignity, threatened by “designing for lawfulness” and by the ensuing lack of control by road users on their own behaviour, would be achieved to a greater extent if they were given control, in a meaningful sense, over fully automated vehicles. As already mentioned, this is not a paradox if we conceive control as in MHC. In the next paragraph, we will provide a very simple and abstract example of what this means in terms of actual design.

### A case scenario: the zebra crossing

As we have seen, to maximize MHC over a system, the two conditions of tracking and tracing have to be maximally satisfied. But how should those notions be operationalized in terms of system design? In the following model of an urban scenario, we will sketch what a simple design for MHC may look like and how it can contribute to minimize some value trade-offs while striving for safety and safe coordination. The case is necessarily sketchy, as a longer and more detailed treatise would require a dedicated paper, but we hope this will help illustrate the content of the previous sections and show the reader the potential in action of the MHC framework.

#### The tracking condition: a zebra crossing case

Consider an urban traffic scenario where multiple agents, both artificial and human, are interacting with each other. In order for the system to remain under MHC, the reasons that move the system, both moral and practical ones, must be clearly identifiable, together with their human carrier(s); this is the tracking condition. Therefore, the next step is designing the system to allow as many as possible of the participating agents to be recognized as the system controllers at any point in time. In order to do that, the system should be able to respond to the reasons of as many as possible of its users. This will maximize overall MHC and, therefore, as seen in the previous sections, minimize the negative impacts raised by the individual coordination strategies.

**Fig. 3:**
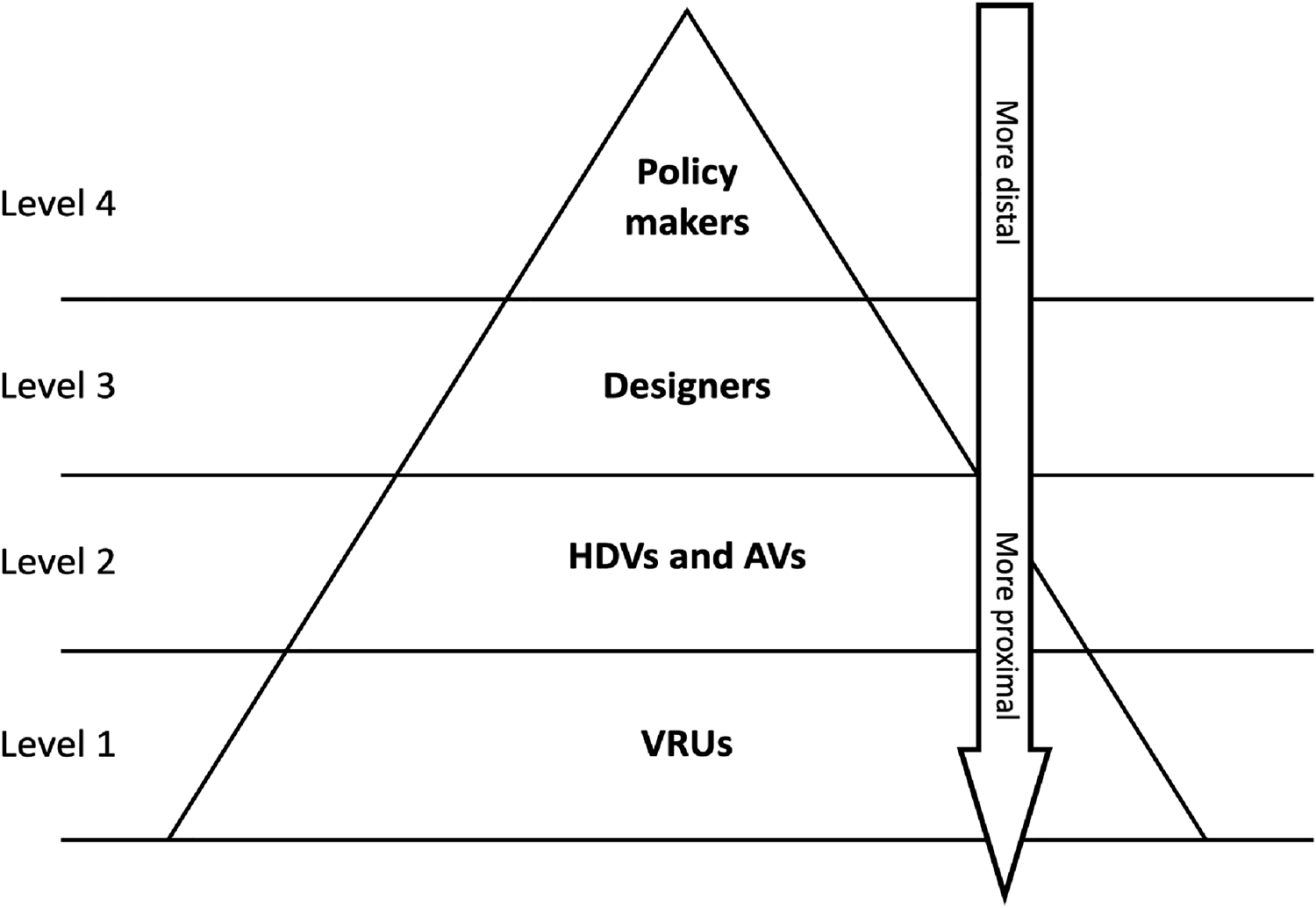
main classes of relevant agents that have a role in determining the behaviour of our example system, a zebra crossing. The vertical arrow indicates the proximity of each class of agents’ reasons to the behaviour of the system

For that reason, we propose a hierarchical scheme, represented by the pyramid of Fig. 3, to which we can overlay some behavioural rule(s) of the system, those that govern whose reasons the system should respond to at different points in time, in a flexible and situation-dependent way. This approach provides a criterion the ultimately allows to dynamically redistribute MHC in a way that recognizes and tracks the interests of the largest possible number of agents at any point in time. In turn, this maximises the benefits of the three strategies, while minimizing their undesired implications.

Figure 3 identifies (arbitrarily, to some extent) the main classes of relevant agents that have a role in determining the behaviour of our example system, a zebra crossing. There, we have an urban road governed by a local road authority (level 4 agent in Fig. 3 below). Partially automated vehicles, which are in part controlled by an automated driving system designed by employees at a vehicle manufacturer (level 3 agents) and in part by their human driver (level 2 agent), make use of this road. At a certain location, a zebra crossing is present on the road for pedestrians and other vulnerable road users (level 1 agents) to cross safely.

Proximal agents, those with a more direct impact on–and more directly impacted by–the system’s behaviour, are positioned at levels 1 and 2 of the pyramid. To protect their freedom, autonomy and human dignity, we have to make the system prioritize as much as possible proximal agents’ reasons (practically all road users, in levels 1 and 2), over those of the distal ones, such as road authorities or government (levels 3 and 4). This is meant to avoid the implications related to the any strand of what we called “designing for lawfulness”, for which potentially draconian measures might be needed to align proximal agent’s behaviour to that of AVs.

HDV drivers will be granted freedom to choose (to some extent) their speed and driving style. Users of AVs will be able to do that too, perhaps selecting among different automated driving styles or settings. Also, VRUs will enjoy freedom of movement and no dystopic behavioural control, while still being mandated to remain within certain legal boundaries. Levels 1 and 2, representing, respectively, vehicles users (from AV’s passengers to HDV’s drivers) and VRUs, are similar in terms of proximity value, but they are placed at different levels in the hierarchy. This is just a normative decision that is not reflected by any measure of proximity. Rather, it is aimed at promoting the interests of VRUs. These road users are not simply the most vulnerable, but they are also more negatively affected by any lack of control, as we have seen while discussing the consequences of trying to adapt them to AVs behaviour. The inherent value of the MHC-driven design we are exemplifying over any of the three strategies singularly taken or just simply combined together, is that attributing (*meaningful human*) control to VRUs does not mean withdrawing it from any other agent in the system, for as long as their interests and values are aligned. The pyramid is meant to provide the necessary categories for a behavioural rule that dynamically maximises MHC across all the users, thereby providing with more circumstantial–and more intelligent–solutions to coordination problems. We propose, as a mere example, the following rule:

*The system’s behaviour (as a whole) should respond to the reasons of the lowest hierarchical level that responds to the reasons of all the higher levels that also respond to the reason of all the higher levels.* 8

According to our rule, the default behaviour of the system is that it responds to level 1. A pedestrian lawfully crosses the street at a zebra crossing. AVs and HDVs should stop, thereby responding to the reasons of lawful drivers and lawful designers, all compliant with a set of more general laws and societal values. If the driver/passenger at level 2 does not want to stop, thereby not complying with the distal reasons of society and policy-makers (higher levels) their control will be withdrawn and the vehicle will reject operational control (where present), and continue to make the interests–and respond to the reasons–of the lower, level 1 road user, who has to be prioritized. We can observe at this point that this design decision does not grant freedom *under every circumstance* to e.g. a driver. In fact, a driver who does not respond to the reasons of a VRU, is not given the freedom to choose to override them and, perhaps, intentionally hit a pedestrian. If, however, a pedestrian at level 1 does not respond to the reasons and interests of the more distal levels, e.g. in case an ill-intentioned pedestrian wants to illegally exploit automated vehicles, the system would start to prioritize the reasons and interests of the users of AVs at the immediately superior level, number 2 in this case. Level 2 agents would be able, for instance, to choose whether to regain operational control and decide the best course of action (in manual or automated modes), still remaining within the technical and legal boundaries established, respectively, at levels 3 and 4. AVs users’ choices might at such point range from displaying discouraging behaviour (e.g. honking) to carefully regain control and manoeuvre around the pedestrian. Alternatively, they could let the AVs default to a certain pre-programmed behaviour. The system, overall, remains always responsive to the reasons of the different agents in different ways. It constantly responds to some of the important values that are expressed at level 4, such as safety and democratic control. In fact, the reckless pedestrian is not harmed by the automated car: that would be prevented and that protection is granted by level 4 responsiveness. At the same time, the system is not responsive to their personal, most proximal intentions, such as that of stalling the traffic. Rather, it prioritizes drivers’ reasons and interests in that case, and let them decide among different possible courses of action. Ultimately, whenever an agent at a certain level entertains reasons and interests that collide with those of any of the higher levels, those reasons are not upheld by the system. It is therefore worth noticing how this system design, rather than merely shifting control and *assigning* it to different users, considers all users as being in control for as long as certain conditions are met, and *withdraws* control when that is no longer the case.

A possible objection would be that a proximal agent, e.g. a VRU, is always interested in HDVs or AVs not speeding, as they may represent a risk for their safety. This would mean that vehicles users would never be able to enjoy the freedom and dignity that were threatened by the “designing for lawfulness” strategy in the first place. However, this objection only considers one value, safety, and disregards any other value, which we assume in our example to be of primary interest of all users at different levels. This means that, despite not all negative value impacts can be eliminated, an optimal compromise could be found in a system that is sufficiently sensitive and responsive to reasons. In practical terms, that could mean that a certain degree of speeding would be granted in the name of freedom in all those areas where other values, e.g. safety, are less at risk. MHC theory provides a conceptual toolbox that facilitates finding optimal compromises between many values and many stakeholders, but it is agnostic to which particular values are at play in a particular system, as well as to which of them *should* be prioritised.

#### The tracing condition in the zebra crossing case

In a system that is designed to maximize tracking, the locus of human control should ideally be always clearly identifiable. A system may be very transparent in terms of retrieving its numerous controllers and the reasons behind each of its behaviours. It could be also a very reactive system, that is always able to align its behaviour with one or another of the participating human agents. However, this does not per se entail that those controllers are “good” controllers. One could indeed influence a system’s behaviour, but without e.g. being aware they are doing so, or what to do when the system misbehaves, or what the consequences of this behaviour might be. One important value, (moral) responsibility, is granted in such system only to the extent another condition for control is realized into the system’s design, the one we called tracing. The tracing condition requires suitable controllers (good carriers of responsibility) to be capable, knowledgeable and aware of their role of controllers in the system. This, in turn, requires systems designers (traffic designers, vehicles designers, regulators…) to make sure the system never responds to the reasons of those components that do not fulfil those requirements. For example, how suitable is a pedestrian to fulfil a major role (which, again, is necessary for safety and accountability) in the control chain? MHC theory is not meant to provide answers to these questions, which remain partially empirical in nature. What the tracing condition can do, however, is to provide traffic psychology and engineering with the normative framework to include a wider range of *desiderata* in their investigation. In simpler terms, it could help considering a wider range of societal values in the enduring investigation on human capacities and urban design.

Fulfilling the tracing condition is not just a matter of empirical investigation and technical design, but also a question of institutional design, because MHC is indirectly promoted by improving citizens’ skills in upholding their role responsibility towards society. This would entail for instance the idea of educating VRUs to interact with mixed traffic urban roads, together with drivers and vehicle users in general. In a way, this is an expected price to pay when control–and the ensuing responsibility–is more evenly distributed across agents of a complex sociotechnical system.

## Conclusions

In this paper, we have presented an alternative perspective on coordination and safety in mixed AV-HDV-VRU traffic. We have claimed that some of the main strategies proposed to address coordination problems might be overly burdening in terms of the value trade-offs they bring about. To address that problem, we suggested that a fourth strategy, inspired by meaningful human control theory, can help to better frame coordination issues and contribute to overcome those trade-offs, ultimately offering a more value preserving approach.

Our concluding remarks revolve around the feasibility of our proposal. One of the main culprits of this proposal regards the level of responsiveness to reasons that the whole sociotechnical system embedding an MHC inspired design should entertain. Whereas human agents are well suited for such responsiveness to contexts and reasons, artificial agents that are part of an ideal MHC designed system are also presumed to be able to discern when parts of the system (including human agents) become unresponsive to certain reasons and values endorsed by other relevant agents. Such sensitivity is key to allow a seamless shift of control throughout the system. On one hand, this remains object for further research, aimed at establishing how such system could and should behave in a number of different scenarios, and what is technically possible. On the other hand, we can already preliminarily observe that achieving adequate responsiveness to reasons and values from an artificial agent does not necessarily represent something exclusively related to technical limitations in the AI performance. This property, in fact, is not to be taken literally, and should not be interpreted as a plea for human-level general AI. Rather, we believe that smart design solutions, e.g. setting up the environment in a certain way, creating clever physical constraints and whatnot, could take us a long way even without tapping into the current AI performance, which is, in any case, quickly moving forward. This idea has been recently investigated in works that are oriented towards the practical implementation of MHC inspired design solutions (see e.g. Calvert et al. 2019; Calvert and Mecacci 2020).

## Data Availability

Data sharing not applicable to this article as no datasets were generated or analysed during the current study.

## Footnotes

1 The most broadly accepted levels of automation are the SAE-levels (SAE 2018), which range from no automation (level 0), through to low level automation with shared operational tasks for a driver and vehicle (levels 1–2), conditional automation during which the driver has a monitoring role, to high automation, which may be considered fully autonomous (levels 4–5).

2 We are talking here of breaking the rules for the sole purpose of imitating human behaviour, versus cases where breaking the rules is legally justified, e.g. when speeding up invading another lane could contribute to avoid an accident.

3 A similar point has been expressed in a recent report by the European Commission Expert Group on driverless mobility (Horizon 2020 Commission Expert Group to advise on specific ethical issues raised by driverless mobility (E03659) 2020).

4 Admittedly, in some way, also machines can be pressed to give explanation for their behavior and be incentivized to not repeat undesirable behavior, this is indeed part of the process of machine learning. However, it is debatable whether this process can be equated to the moral and social learning involved in public deliberation (see e.g. Stilgoe 2018).

5 Compare Santoni de Sio (2017).

6 The term “system” here is used to denote a “sociotechnical system”, an arbitrary set of human and non-human agents, together with technological infrastructures, that are relevant for a certain control problem. In our case, this will be urban traffic, thereby including traffic infrastructure, all road users, traffic regulators, vehicle designers and so forth.

7 A more detailed account of the different kinds of reasons and intentions according to classic philosophy of action can be found in (Mecacci AND Santoni de Sio 2020).

8 This exemplificatory rule may seem similar to other algorithms proposed in the literature to deal with morally loaded decisions, such as the trolley problem. However, this rule operates at a meta-level, if compared to other proposed behavioral algorithms. In fact, rather than committing to a certain set of values, or a certain ethical theory, to control the behaviour of the system, it tells us how the system should “track” those values in the relevant stakeholders while remaining neutral to their specific values. This does not mean that the strategy does not promote values. On the contrary, by allowing Meaningful Human Control, it allows controllers values, whichever they may be, to be better expressed in the system design and operation.

## References

[CR1] Botvinick MM, Rosen ZB (2009). Anticipation of cognitive demand during decision-making. Psychol Res.

[CR2] Braithwaite I, Callender T, Bullock M, Aldridge RW (2020). Automated and partly automated contact tracing: a systematic review to inform the control of COVID-19. Lancet Digit Health.

[CR3] Bratman ME (1987). Intentions, plans and practical reason.

[CR4] Calvert SC, Mecacci G (2020). A conceptual control system description of cooperative and automated driving in mixed urban traffic with meaningful human control for design and evaluation. IEEE Open J Intell Transp Syst.

[CR5] Calvert SC, Soekroella A, Wilmink I, Van Arem B (2016) Considering knowledge gaps for automated driving in conventional traffic. In: Fourth international conference on advances in civil, structural and environmental engineering—ACSEE 2016, December. 10.15224/978-1-63248-114-6-33

[CR6] Calvert SC, Heikoop DD, Mecacci G, van Arem B (2019). A human centric framework for the analysis of automated driving systems based on meaningful human control. Theor Issues Ergon Sci.

[CR7] Calvert SC, Mecacci G, van Arem B, Santoni de Sio F, Heikoop DD, Hagenzieker M (2020). Gaps in the control of automated vehicles on roads. IEEE Intell Transp Syst Mag.

[CR8] Campbell M, Egerstedt M, How JP, Murray RM (2010). Autonomous driving in urban environments: approaches, lessons and challenges. Philos Trans R Soc A Math Phys Eng Sci.

[CR57] Carsten O, Martens MH (2019) How can humans understand their automated cars? HMI principles, problems and solutions. Cogn Tech Work 21:3–20. 10.1007/s10111-018-0484-0

[CR9] Danaher J (2016). Robots, law and the retribution gap. Ethics Inf Technol.

[CR10] Eriksson A, Stanton NA (2017). Takeover time in highly automated vehicles: noncritical transitions to and from manual control. Hum Fact J Hum Fact Ergon Soc.

[CR11] Fischer JM, Ravizza M (1998). Responsibility and control: a theory of moral responsibility.

[CR12] Flemisch F, Abbink D, Itoh M, Pacaux-Lemoine M-P (2019). Special issue on shared and cooperative control. Cogn Technol Work.

[CR13] Gerdes JC, Thornton SM (2016). Implementable ethics for autonomous vehicles. autonomous driving.

[CR14] Haselager P, van Dijk J, van Rooij I (2008). A lazy brain? Embodied embedded cognition and cognitive neuroscience. Handbook of cognitive science.

[CR15] Heikoop DD, de Winter JCF, van Arem B, Stanton NA (2018). Effects of mental demands on situation awareness during platooning: a driving simulator study. Transp Res f Traffic Psychol Behav.

[CR16] Horizon 2020 Commission Expert Group to advise on specific ethical issues raised by driverless mobility (E03659) (2020) Ethics of connected and automated vehicles: recommendations on road safety, privacy,fairness, explainability and responsibility. 10.2777/035239

[CR17] Horowitz MC, Scharre P (2015) Meaningful human control in weapons systems: a primer

[CR18] Kayukawa S, Higuchi K, Guerreiro J, Morishima S, Sato Y, Kitani K, Asakawa C (2019) BBEEP: a sonic collision avoidance system for blind travellers and nearby pedestrians. In: Conference on human factors in computing systems—proceedings, p 1–12. 10.1145/3290605.3300282

[CR19] Loh W, Misselhorn C (2019). Autonomous driving and perverse incentives. Philos Technol.

[CR20] Louw T, Kountouriotis G, Carsten O, Merat N (2015) Driver inattention during vehicle automation: how does driver engagement affect resumption of control? In: 4th International conference on driver distraction and inattention (DDI2015), proceedings, Sydney

[CR21] Matthias A (2004). The responsibility gap: ascribing responsibility for the actions of learning automata. Ethics Inf Technol.

[CR22] Mecacci G, Santoni de Sio F (2020). Meaningful human control as reason-responsiveness: the case of dual-mode vehicles. Ethics Inf Technol.

[CR23] Merat N, Jamson AH, Lai FCH, Daly M, Carsten OMJ (2014). Transition to manual: Driver behaviour when resuming control from a highly automated vehicle. Transport Res f Traffic Psychol Behav.

[CR24] Michon JA, Evans L, Schwing RC (1985). Human behavior and traffic safety. Human behavior and traffic safety.

[CR25] Millard-Ball A (2018). Pedestrians, autonomous vehicles, and cities. J Plan Educ Res.

[CR26] Mindell DA (2015). Our robots, ourselves: robotics and the myths of autonomy.

[CR27] Montuwy A, Cahour B, Dommes A (2019). using sensory wearable devices to navigate the city: effectiveness and user experience in older pedestrians. Multimodal Technol Interact.

[CR28] Moyes R (2016) Key elements of meaningful human control. Article 36. http://www.article36.org/wp-content/uploads/2016/04/MHC-2016-FINAL.pdf

[CR29] Nuñez Velasco P, Farah H, van Arem B, Hagenzieker M (2018) WEpod WElly in Delft: pedestrians’ crossing behavior when interacting with automated vehicles using Virtual Reality. In: Proceeedings of the 15th international conference on travel behaviour research, Santa Barbara, USA. http://www.iatbr2018.org/accepted-abstracts.html

[CR30] Nyholm S, Smids J (2020). Automated cars meet human drivers: responsible human-robot coordination and the ethics of mixed traffic. Ethics Inf Technol.

[CR31] Pacaux-Lemoine M-P, Flemisch F (2019). Layers of shared and cooperative control, assistance, and automation. Cogn Technol Work.

[CR32] Parasuraman R, Riley V (1997). Humans and automation: use, misuse, disuse, abuse. Hum Fact J Hum Fact Ergon Soc.

[CR33] Pasquale F (2020). New laws of robotics: defending human expertise in the age of AI.

[CR34] Pettit P (1999) Republicanism: a theory of freedom and government. Oxford University Press, Oxford. https://books.google.nl/books?id=AOfYtIyWOZsC

[CR35] Raz J (1975). Reasons for action, decisions and norms. Mind.

[CR36] Regan MA, Lee JD, Young K (2008). Driver distraction: theory, effects, and mitigation.

[CR37] Reiman JH (1995). Driving to the panopticon: a philosophical exploration of the risks to privacy posed by the highway technology of the future—Santa Clara symposium on privacy and IVHS. Santa Clara Comput High Technol Law J.

[CR58] SAE J3016 (2018) Taxonomy and Definitions for Terms Related to On-Road Motor Vehicle Automated Driving Systems

[CR38] Santoni de Sio F (2017). Killing by autonomous vehicles and the legal doctrine of necessity. Ethical Theory Moral Pract.

[CR39] Santoni de Sio F, Mecacci G (2021). Four responsibility gaps with artificial intelligence: why they matter and how to address them. Philos Technol.

[CR40] Santoni de Sio F, van den Hoven J (2018). Meaningful human control over autonomous systems: a philosophical account. Front Robot AI.

[CR41] Sayer J, Mefford L, Shirkey K, Lants J (2005) Driver distraction: a naturalistic observation of secondary behaviors with the use of driver assistance systems. In: Third international driving symposium on human factors in driver assessment, training and vehicle design, p 262–268

[CR42] Schwarz E (2018) The (im)possibility of meaningful human control for lethal autonomous weapon systems. https://blogs.icrc.org/law-and-policy/2018/08/29/im-possibility-meaningful-human-control-lethal-autonomous-weapon-systems/

[CR43] Seeber KG (2011). Cognitive load in simultaneous interpreting. Interpret Int J Res Pract Interpret.

[CR44] Smids J (2018). The moral case for intelligent speed adaptation. J Appl Philos.

[CR45] Stanton NA, Marsden P (1996). From fly-by-wire to drive-by-wire: safety implications of automation in vehicles. Saf Sci.

[CR46] Stilgoe J (2018). Machine learning, social learning and the governance of self-driving cars. Soc Stud Sci.

[CR59] Tillema T, Gelauff G, van der Waard J, Berveling J, Moorman S (2017) Paths to a Self-Driving Future, Five Transition Steps Identified, KiM, Netherlands Institute for Transport Policy Analysis

[CR47] Van den Brule R, Bijlstra G, Dotsch R, Haselager P, Wigboldus DHJ (2016). Warning signals for poor performance improve human-robot interaction. J Hum Robot Interact.

[CR48] Van den Hoven J, Lokhorst G-J, Van de Poel I (2012). Engineering and the problem of moral overload. Sci Eng Ethics.

[CR49] van den Hoven J, Owen R, Bessant J, Heintz M (2013). Value sensitive design and responsible innovation. Responsible innovation.

[CR50] van Loon RJ, Martens MH (2015). Automated driving and its effect on the safety ecosystem: how do compatibility issues affect the transition period?. Procedia Manuf.

[CR51] Varotto SF, Farah H, Toledo T, van Arem B, Hoogendoorn SP (2018). Modelling decisions of control transitions and target speed regulations in full-range adaptive cruise control based on risk allostasis theory. Transp Res Part Methodol.

[CR52] Velasco N, Farah P, Van Arem H, Hagenzieker B (2017) Interactions between vulnerable road users and automated vehicles: a synthesis of literature and framework for future research. In: Proceedings of the road safety and simulation international conference 2017, p 16–19. https://repository.tudelft.nl/islandora/object/uuid%3A7f297ea5-34bf-4a88-a62e-b98523bbf9ae

[CR60] Vlakveld W (2016) Developed Nations. In: Fisher DL, Caird JK, Horrey WJ, Trick LM (eds) Handbook of Teen and Novice Drivers; Research, Practice, Policy, and Directions. Boca Raton, CRC Press

[CR53] Wenzl R (2018) The kill chain: inside the unit that tracks targets for US drone wars|World news|. The Guardian. https://www.theguardian.com/world/2018/jan/23/the-kill-chain-inside-the-unit-that-tracks-targets-for-us-drone-wars

[CR54] Wolf I (2016). The interaction between humans and autonomous agents. Autonomous driving.

[CR55] Young KL, Salmon PM, Cornelissen M (2013). Missing links? The effects of distraction on driver situation awareness. Saf Sci.

[CR56] Zeeb K, Buchner A, Schrauf M (2015). What determines the take-over time? An integrated model approach of driver take-over after automated driving. Accid Anal Prev.

